# Benefits of vitamin D supplementation to attenuate TBI secondary injury?

**DOI:** 10.1515/tnsci-2020-0195

**Published:** 2021-12-15

**Authors:** Kiana Saadatmand, Saba Khan, Quaratulain Hassan, Raymond Hautamaki, Rani Ashouri, Josh Lua, Sylvain Doré

**Affiliations:** Department of Anesthesiology, Center for Translational Research in Neurodegenerative Disease, University of Florida College of Medicine, Gainesville, FL, 32610, United States of America; Departments of Psychiatry, Pharmaceutics, Psychology, and Neuroscience, McKnight Brain Institute, University of Florida College of Medicine, Gainesville, FL, 32610, United States of America

**Keywords:** calcidiol, calcitriol, concussion, traumatic brain injury, vitamin D receptor

## Abstract

Vitamin D supplementation has been shown to improve outcomes for patients suffering from a variety of illnesses such as stroke and cancer. Vitamin D deficiencies have been associated with longer hospital stays, greater severity of symptoms, and death in some complex cases. Due to vitamin D’s burgeoning role in improving patient outcomes, a new sector of research is focusing on the lesser-known implications of vitamin D on health. Traumatic brain injury (TBI) affects approximately 69 million people worldwide per year. Here, we summarize the current scientific understanding of vitamin D dynamics with TBI to elucidate a potential way to lessen the cascade of secondary damage after an initial insult, with the goal of improving overall patient outcomes. Because vitamin D supplementation has been correlated with better outcomes in other pathologies involving immune and inflammatory molecules, it is important to study the potential effect of vitamin D deficiency (VDD) and supplementation on TBI outcomes. Research on vitamin D supplementation in TBI remains in the preliminary stages. There is still much to learn about vitamin D deficiency, dosage, variants of supplementary forms, mechanisms, and its role in TBI.

## 1 Introduction

### 1.1 Vitamin D physiological overview

**Figure 1:**
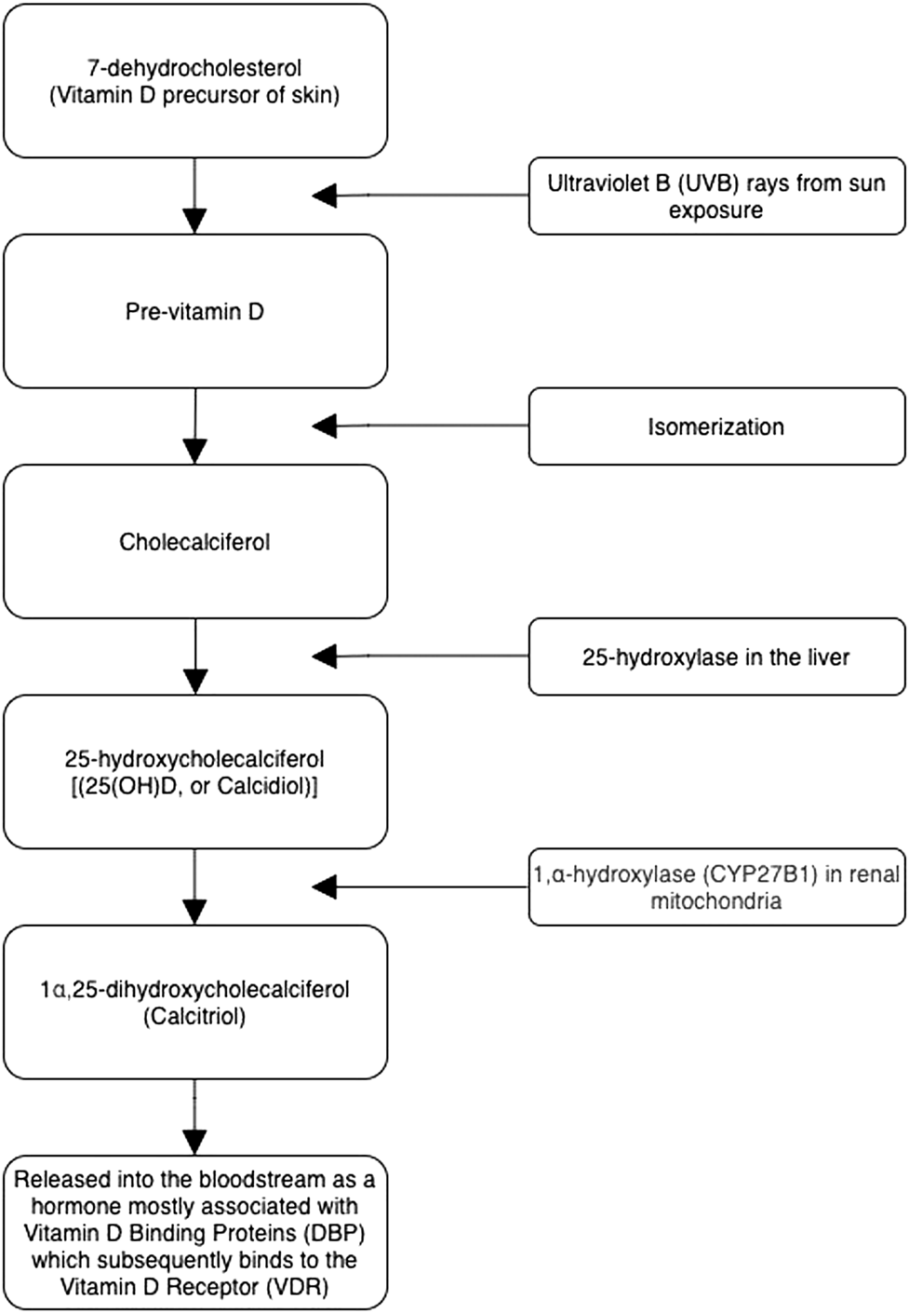
The synthesis of vitamin D.

The term vitamin D encompasses a group of fat-soluble secosteroids responsible for increasing intestinal absorption of magnesium, phosphate, and calcium [1]. For humans, the primary source of vitamin D is the ultraviolet B light in sunlight, which causes 7-dehydrocholesterol to photolyze to previtamin D in the epidermis [2]. Previtamin D, an intermediate, is converted to cholecalciferol through isomerization and is enzymatically (via 25-hydroxylase in the liver) converted to 25-hydroxycholecalciferol [25(OH)D, or calcidiol] [2]. Calcidiol is the circulating and active form of vitamin D measured in serum. The best measurement of vitamin D status is calcidiol, which has a half-life of approximately 2 weeks [3]. Calcidiol is further modified by mitochondrial enzyme 1,α-hydroxylase (CYP27B1) to yield the vitamin D active form, calcitriol (1α,25-dihydroxycholecalciferol), in the renal mitochondria. After calcitriol is released into the bloodstream, it binds to the vitamin D binding proteins (DBP). Once the hormone is released, it is able to bind to a vitamin D response element and act as a transcription factor [4]. Calcitriol binds to the vitamin D receptor (VDR), which binds to a vitamin D response element in the promoter region of regulated genes [4]. Calcitriol can also bind to cell membrane receptors for rapid nongenomic signaling (Figure 1).

The classical function of this hormone is to control calcium levels in the blood by regulating gene expression of renal excretion, intestinal Ca^2+^ absorption, and movement in and out of bones. Recent studies have revealed that the role of vitamin D goes beyond bone metabolism. The noncalcemic effects include modulation of apoptotic activity, immunomodulatory (in dendritic cells, macrophages, and lymphocytes), and neuroprotective functions [5]. These newly discovered functions might be correlated with the presence of VDRs in various organs and cell types. Studies show an inverse correlation between the concentration of markers of inflammation, such as pro-inflammatory cytokines and chemokines, and vitamin D levels, which might suggest a link between the inflammatory response and insufficient vitamin D levels [6]. Studies have linked vitamin D deficiency (VDD) to higher infection rates, higher mortality rates, a longer length of hospital stay, and a longer duration of mechanical ventilation [6].

Vitamin D is obtained through cutaneous synthesis by sunlight (80–90%) and nutritional means (10–20%) [7]. Two common variants of this secosteroid are vitamin D_2_ (ergocalciferol) or D_3_ (cholecalciferol). Vitamin D_2_ is found in some plants and yeast and is a less active form of vitamin D_3_. Milk and some cereals are major sources of vitamin D_3_. Fatty fish, such as mackerel and salmon, also have vitamin D_3_. For dietary supplements, vitamin D_3_ supplementation in humans is usually more effective at raising serum vitamin D than vitamin D_2_ [8].

VDD, which is medically diagnosed as serum levels lower than 20 ng/mL (50 nmol/L) 25(OH)D_3_, can lead to obesity, hypertension, and even cancer [9]. Vitamin D insufficiency (VDI) is the calcitriol concentration at which a subclinical deficiency exists. Currently, the National Academy of Sciences has several cutoffs corresponding to different populations ranging from 37.5 nmol/L (15 ng/mL) to 77.5 nmol/L (31 ng/mL) [4]. Experts have recommended that these cutoff values could be increased.

#### 1.1.1 TBI

Traumatic brain injury (TBI), an event in which an external force injures the brain, is the most common trauma globally and is one of the leading causes of disability and injury-related death among individuals aged 40 years or younger [10]. In the United States, 1.5 million people experience head trauma annually, and the annual economic cost exceeds $56 billion [11]. Clinically, TBI incidence is generally categorized by whether it is a closed head or open head injury. A person with acute TBI can exhibit many effects such as altered information processing, memory deficits, reduced psychomotor ability, cognitive deficits with altered general fluid intelligence, attention deficit, changed hearing, difficulty in understanding language, and altered visual-spatial skills. The primary treatment protocol for a brain injury includes adequate oxygen delivery, limiting the initial trauma, inhibiting secondary insult due to hypotension and hypoxia, and sufficient metabolic substrates [8].

TBI consists of primary and secondary damage. Primary damage is an immediate injury due to mechanical factors in various forms, such as surface contusions, intracranial hemorrhage, and diffuse axonal injury. The secondary injuries are delayed complications that do not clinically present themselves for hours to days after the primary injury occurs. The delayed appearance of the secondary damage provides a window of opportunity to treat such outcomes. The secondary injury includes damage due to ischemia, brain edema, and changes in neuronal function [12]. During the post-injury period, a few anatomical alterations in the body, such as inflammatory biomarkers, can determine the severity and outcome of TBI. For instance, the level of cytokines, such as interleukin (IL) 1β, IL-6, and tissue necrosis factor α (TNFα), increase after a severe injury and can be useful as an indication of the extent of systemic inflammation. Prior observations show that an increase in IL-6 levels in an acute period after TBI is a key indicator for severe complications [1]. Although there have been advances in research and development, healthcare costs correlated with TBI continue to increase substantially. Thus, there is a need for research focused on novel pharmacological strategies that effectively modulate TBI secondary injury pathways [13].

### 1.2 Relevance of vitamin D to the pathophysiology of TBI

Because vitamin D is involved with regulating calcium levels and has recently been shown to regulate proliferative and apoptotic activity and play an immunomodulatory role, vitamin D supplementation in the acute phase of the injury may attenuate the secondary injury [1]. Vitamin D also supports neural protection within the central nervous system (CNS) through antioxidant mechanisms, enhanced nerve conduction, and detoxification mechanisms [14]. Most studies investigating a role for vitamin D supplementation after brain injury do so in combination with progesterone (PROG) [8].

Studies on vitamin D effects on patients with TBI have not been adequate and have not arrived at a definitive conclusion [10]. This review summarizes the literature on the association of vitamin D with TBI to highlight the promises and limitations of supplementation in TBI treatment for potential future experimental research.

## 2 Biochemistry

### 2.1 Vitamin D interactions with the CNS, blood–brain barrier, immune and vascular systems

Vitamin D plays a significant role in maintaining general health. A glycoprotein called osteopontin (OPN) that is associated with vitamin D has been demonstrated to have neuroprotective effects for ischemic injuries that involve the brain and other organs [15]. In a study assessing vitamin D’s ability to attenuate disruptions to the blood–brain barrier (BBB), it was found that intranasal administration of vitamin D_3_ upregulates OPN in astrocytes, protecting against BBB disruption [15].

In addition to the neuroprotective effects of vitamin D via upregulation of OPN, vitamin D has been demonstrated to improve COVID-19 outcomes. A study analyzing the impact of short-term high-dose vitamin D supplementation (60,000 IU of cholecalciferol) on patients with COVID-19 without comorbidities found that a greater proportion of patients who received vitamin D treatment obtained COVID-19 negativity sooner than those without treatment [16]. Additionally, the study provided evidence of vitamin D’s anti-inflammatory properties. Patients who received a high dosage of cholecalciferol had a significant decrease in fibrinogen, an inflammatory marker associated with COVID-19 [16]. Of patients who were given 266 mcg of 25(OH)D, 2% required admission to the intensive care unit (ICU), whereas 50% of patients excluded from the treatment were taken to the ICU [17]. Another study reported that nursing home patients with COVID-19 who were given bolus vitamin D had a survival rate of 82.5%. In comparison, COVID-19 patients who were not given vitamin D had a survival rate of 44.4% [18].

VDD and VDI are associated with higher risks for cardiovascular disease. Vitamin D can promote vasodilation, relieve arterial pressure, and enhance post-stroke blood flow to neurons by activating nitric oxide synthase (NOS) pathways. Measuring the vessel diameter and endothelial functions of the basilar artery have shown vitamin D’s anti-inflammatory effects and attenuation of cerebral vasospasm [9]. Individuals with VDD were discovered to have higher rates of angina, myocardial infarction, and heart failure, as well as more cardiovascular risk factors, including hypertension, atherosclerosis, and diabetes [19]. VDD can result in inflammation of systemic and vascular tissues, as well as an increase in vascular stiffness and platelet volume [20]. Vitamin D helps in modulating the growth of cardiomyocytes and smooth muscle cells. Furthermore, vitamin D has been shown to act as an antihypertensive agent. A study analyzing the effect of vitamin D on cardiovascular health demonstrated that VDR^−/−^ mice exhibited hypertension, cardiac hypertrophy, and increased activation of the renin–angiotensin–aldosterone system, whereas mice given injections of 1,25(OH)_2_D_3_ exhibited suppressed activation of the renin–angiotensin–aldosterone system [19,21]. Vascular smooth muscle cells and endothelial cells have VDRs that convert calcidiol to calcitriol. Despite this, vitamin D has also been shown to prevent the growth of smooth vascular cells due to the inflow of calcium into the cell, which increases the calcification of smooth cells [20]. Because smooth cells play an active role in calcification and are contractile, the calcification driven by vitamin D causes the cells to work less effectively, leading to complications and possible mortality [22]. These complications include atherosclerotic plaque rupture, myocardial infarction, stroke, atherosclerosis, hyperlipidemia, cell death, and inflammation [22].

Studies have also shown a link between vitamin D and neuroinflammation. In response to TBI, astrocytes and microglia are activated, releasing inflammatory mediators in the brain [23]. Although the microglia assist in the promotion of neurological recovery, microglia can produce an excessive amount of pro-inflammatory mediators, which can exacerbate brain damage, hindering the brain’s ability to repair itself [23]. TBI results in an increase of the pro-inflammatory cytokines IL-6, IL-17, IL-1β, IFNγ, and TNFα and a decrease in the anti-inflammatory cytokines TGF-β1, IL-4, IL-10, and IL-13 [23]. A study examining the association between vitamin D and inflammatory activity found that vitamin D lowered the pro-inflammatory cytokines IL-1β, IL-12, IL-6, TNFα, and IFNγ, which demonstrates its ability to mitigate neuroinflammation associated with TBI through its activation of anti-inflammatory markers [24]. Another study noted that patients with TBI who had insufficient vitamin D levels, i.e., lower than 20 ng/mL, were marked by a greater presence of neuroinflammation than those who had adequate vitamin D levels [10]. Neuroinflammation was measured by assessing the change in the following inflammatory markers: IL-6, C-reactive protein, and the monocyte protein-1 (MCP-1) [10].

Vitamin D_3_ is able to bind and stimulate nerve growth factors, which are secreted proteins that aim to help with the survival and differentiation of neurons and assist in neuroprotection after injury [4]. Some notable neurotrophins impacted by vitamin D_3_ supplementation are nerve growth factor (NGF), neurotrophin-4 (NT-4), brain-derived neurotrophic factor (BDNF), and glial-derived neurotrophic factor (GDNF) [25]. Both NGF and BDNF are needed for glial survival and remyelination, whereas NT-4 and GDNF are used for regeneration and recovery after CNS injury. One study has reproted that vitamin D hormone (VDH) pretreatment for 8 days could increase GDNF and in turn attenuate cortical infarction induced by middle cerebral artery ligation in rats, demonstrating vitamin D’s ability to combat neuroinflammation [24]. The release of nitric oxide, a mechanism involved with brain damage that contributes to neuronal damage, has been shown to decrease with the supplementation of vitamin D_3_ or potentially affect the induction of GDNF [26].

### 2.2 Current clinical perspective on vitamin D

It is estimated that at least half of the world’s population has VDI, and approximately 1 billion people suffer from VDD. VDI and VDD are most common in South Asia, Australia, the Middle East, and South America [27]. The prevalence of VDD and VDI can be attributed to various factors, including diet, lifestyle, location, climate, and others. Although it is hard to influence these factors, VDI and VDD can be combatted through vitamin D supplementation. However, despite the ability of vitamin D supplementation to mitigate low vitamin D levels, the number of people in the world suffering from VDD and VDI remains essentially unchanged.

The recommended dietary allowance for vitamin D is 600 IU (15 mcg) for adults and children. The amount is slightly higher for older adults, at 800 IU (20 mcg). When it comes to vitamin D allowance, there is no set amount. Recommendations depend on a variety of factors such as age, pre-existing conditions, lifestyle, exposure to sunlight, diet, genetics, and others [27]. People with higher concentrations of melanin and older people have a more challenging time producing vitamin D through sunlight; therefore, they require a larger volume of supplementation. For example, a younger patient with a lighter skin tone needs approximately 4 min of ultraviolet B exposure to 25% of their body (legs and arms); however, individuals who are older or have a darker skin tone require 18 min to obtain 1,000 IU of vitamin D_3_ [28]. Exposure to sunlight varies depending on the season, geographical location, and lifestyle choices. People who regularly wear sunscreen, stay indoors, and live in places with greater cloud coverage may be at risk for VDD.

The daily recommended dose of vitamin D ranges from 400 to 2,000 IU. For infants, 400 IU is recommended, and for children, adolescents, and adults, 600 IU is recommended [29]. The largest dose, 800 IU, is recommended for adults over 70 years old. Vitamin D dosages can become toxic when they go over the recommended dosages (400–2,000 IU/day), which are dependent on disease state, age, ethnicity, and body weight. Vitamin D toxicity can cause hypercalcemia and hypercalciuria, which, in turn, can induce symptoms including nausea, vomiting, muscle weakness, pain, neuropsychiatric disturbances, polyuria, and kidney stones. In extreme cases, toxicity can result in renal failure, cardiac arrhythmias, calcification of soft tissues, and death. The limit is 2,000 IU/day for infants, whereas for children, adolescents, and adults, the limit is 4,000 IU. The level of intake that induces toxic effects is a matter of debate, however, with studies finding the minimum level of vitamin D toxicity to be as low as 4,000 IU/day and as high as 10,000 IU/day [29]. However, these instances are rare and usually connected to some form of vitamin D hypersensitivity such as Williams–Beuren syndrome, and the most common threshold for vitamin D toxicity is around 50,000 IU/day in most studies reviewed by the NAM (formerly the IoM) in 2011 [30]. However, it is important to point out that recommendations and daily allowances can vary widely depending on environmental, genetic, and social factors.

Furthermore, these upper limits are for daily intake, and the upper limit for a single dose of vitamin D is unknown. A review of single, high-dose vitamin D administration for patients with VDD found that dosages of 100,000–300,000 IU were well-tolerated [31], though it should be noted that most of these patients were older adults and had outside of normal physiological concentrations of vitamin D and calcium due to their VDD. One case study by Klontz and Acheson found that a single dose of ∼1,131,840 IU induced vitamin D toxicity in a 58-year-old woman with diabetes mellitus and rheumatoid arthritis [32], which was the lowest dosage reported by the NAM in its 2011 review [30]. Research still needs to be done on recommended dosages of vitamin D to maximize patient outcomes. Currently, research is being done to assess the efficacy of high-dose and low-dose vitamin D supplementation on mortality rates and inflammatory factors of patients suffering from a wide range of conditions, including TBI and COVID-19.

## 3 Correlation between vitamin D and TBI

The crucial role of VDH in brain function was highlighted after discovering high-affinity VDRs in the brains of animal models and was confirmed by identifying VDH metabolites in the cerebral spinal fluid of healthy patients. Animal studies have shown that the VDR is located within particular brain regions, including the hypothalamus, cortex, hippocampus, amygdala, thalamus, and cerebellum [33]. Initially, it was suggested that VDH levels in the CNS were dependent on the passive and active transport of the hormone across the BBB. However, since discovering 25-hydroxylase and 1α-hydroxylase enzymes in the human brain, it has instead been proposed that there is local bioactivation of VDH in the CNS. The role of vitamin D in neuroprotection is multidimensional. Vitamin D has been shown to lessen the inflammatory response after TBI, which mitigates neuronal injury, decreases neuronal cell death, and improves the CNS functional outcome. VDH also minimizes neuronal influx of calcium and the release of excitotoxic glutamate, which stimulates cell death after TBI. Vitamin D lowers intracellular calcium levels by downregulation of L-type voltage-sensitive Ca^2+^ channels and upregulation of intracellular Ca^2+^ buffering, leading to decreased glutamate release and preventing neurotoxicity. In addition, through increasing intracellular glutathione, an antioxidant, vitamin D increases the rate of free radical scavenging and reduces oxidation. Vitamin D can improve the protection of microtubules and regeneration of the axonal and neuronal cytoskeleton, which promote axogenesis and an increase in axon diameter in injured axons resulting from TBI. Additionally, vitamin D upregulates neurotrophic growth factors and other markers that play a role in neuronal function, development, and survival [8].

Vitamin D can induce anti-inflammatory cytokines and various antimicrobial proteins, such as cathelicidin, by neutrophils and macrophages. Such proteins play a significant role in protecting the integumentary barrier sites from infections. Infectious diseases such as sepsis and pneumonia are prevalent in patients with TBI, leading to a death rate exceeding 20% [10]. Therefore, in addition to the neuroprotective effects of vitamin D in the CNS, it also could improve TBI outcomes via these antimicrobial factors. Also, VDD is common in patients who have had lengthy post-TBI hospitalizations, which are also linked to complications such as coma, critical illness polyneuropathy, and prolonged recovery.

### 3.1 Pathophysiology of TBI ameliorated by vitamin D

The mechanism by which vitamin D ameliorates post-TBI patient outcomes is not fully understood; however, some studies aim to elucidate relevant pathways as to how vitamin D regulates brain development, cell-cycle control immune regulation, neurological function, inflammation, and cell death. Vitamin D has been described to activate a VDR, which leads to the activation of various proteins, including the A and C isomers of OPN [15]. The upregulation of OPN protects the BBB by CD44 splicing. P-gp glycosylation in endothelial cells and blockage of the VDR and OPN resulted in decreased glycosylated P-gp in the brain [15]. The method by which OPN protects the BBB involves the activation of MAPK phosphatase and MKP-1 in astrocytes and endothelial cells, as well as the phosphorylation of Akt, p42, and p44 and upregulation of GDNF [15]. When examining the pathophysiology by which vitamin D interacts with GDNF to improve post-TBI outcomes, it has been discovered that GDNF protects dopaminergic (DA) neurons against MPP + (1-methyl-4-phenylpyridinium)-induced toxicity by activation of antioxidant enzyme systems, preventing the neurotoxin from depleting ATP from cells [34]. Additionally, the study hypothesized that GDNF would upregulate receptor α1 and c-Ret (signal transducer), which in turn activates a neuroprotective mechanism [34]. Although GDNF was highlighted in the previous study, another study stated that NGF, an alternative nerve growth factor, was involved in the mechanism through which vitamin D improved post-TBI outcomes [35]. Another theory postulates that OPN is protective by inhibiting caspase-3 cleavage using FAK, PI3K, and Akt [15]. The cleavage of CD44 is regulated through the activation of metalloprotease, PKC, Ca^2+^, and Ras oncoprotein [15]. In contrast, another study described a different pathway by which vitamin D improved post-TBI outcomes. Specifically, vitamin D regulates L-type voltage-sensitive Ca^2+^ channels in peripheral tissues, managing brain aging and neuronal vulnerability [1].

### 3.2 Preclinical investigations

Many preclinical studies have demonstrated the positive effects of vitamin D on TBI and the associated neuroinflammation. One experiment that examined the impact of VDD on neuroprotection in aged rats found that VDH deficiency exacerbated TBI symptoms. The deficient rats bled longer, displayed softer bone structure, were less vitally stable during the surgery, and took longer to recover after surgery, indicating that sufficient vitamin D levels are significant in treating TBI. VDD also increased the baseline inflammation in these rats, potentially establishing a detrimental underlying condition. At 72 h after surgery, all inflammatory markers (TNFα, IL-6, NFκB p65, cleaved caspase-3), except for IL-1β and COX-2, were significantly higher in vehicle-treated VDD rodents than in rodents with normal vitamin D levels [36].

In another trial, which demonstrated vitamin D’s ability to combat neuroinflammation, 160 adult male rats were placed in four groups, a TBI model, a sham model, a TBI group with calcitriol treatment, or a sham with calcitriol treatment. This study observed elevated VDR protein expression at the 1-, 3-, and 7-day mark compared with the TBI group (*p* < 0.05), demonstrating that calcitriol activates VDR expression in the TBI rats’ hippocampus CA1 region. It was found that calcitriol significantly lessened the neurological deficits in the rats at the 3-, 7-, and 14-day mark compared to the TBI group (*p* < 0.01) [12]. This study also examined the activity of the enzyme nicotinamide adenine dinucleotide phosphate oxidase (NOX_2_), which is expressed in the hippocampus and promotes cell death and functional impairments post-TBI [37,38]. Nicotinamide adenine dinucleotide phosphate oxidase has various subunits that have different isoforms of NOX_1–5_. Calcitriol treatment significantly reduced NOX activity compared with the TBI group (*p* < 0.01). The expression of NOX_2_ was also evaluated. Rats undergoing the calcitriol treatment showed lower expression of NOX_2_ at the 1-, 3-, and 7-day mark than rats in the TBI group (*p* < 0.01). In the calcitriol group, the cell death rate in the hippocampus CA1 region was significantly lower than in the TBI group (*p* < 0.01). Calcitriol reduced the expression of NOX_2_ in the hippocampus CA1 region and had a cytoprotective effect against neuronal death [12]. These findings suggest that calcitriol can reduce the secondary damage after TBI and might have a role in modulating the activity of NADPH oxidase.

In addition to reducing neuroinflammation secondary to TBI, vitamin D supplementation was shown to alleviate disruptions to the BBB and abate brain edema. As brain edema is a main cause of death in hospitals for people with brain lesions and disruptions to the BBB can lead to neuronal death, ameliorating these secondary effects is imperative to treating TBI. In the study, rats were separated into sham, TBI vehicle, and TBI vitamin D_3_ groups. Cholecalciferol was injected at the following doses: 1, 2, and 5 μg/kg/day. It was shown that the two higher doses of vitamin D (2 and 5 μg/kg/day) were better able to decrease brain edema (*p* < 0.05) and that the highest dose of vitamin D_3_ (5 μg/kg/day) worked best to reduce impairments to the BBB (*p* < 0.01) [39,40,41]. These results illustrate a different protective effect of vitamin D against TBI and support the argument for vitamin D supplementation improving outcomes post-TBI.

To further these discoveries and determine calcitriol’s effect on neurological functions after TBI, 85 male rats were designated to sham, TBI model, or calcitriol treatment groups. This study used various methods to examine the calcitriol treatment effect. VDR expression in the rat cortex of the calcitriol-treated group was higher than in the untreated groups 1–7 days after TBI (*p* < 0.05). In the calcitriol treatment group, the apoptotic cell rate in the rat cortex was significantly lower than in the TBI group (*p* < 0.05). The microtubule-associated protein 1 light chain 3 ratio (LC3II/LC3I ratio) and p62 protein expression in the cortex were also examined. LC3 is located in the autophagosomal membrane and is a marker to assess autophagy activity. LC3I (the soluble form of LC3) was converted to LC3II, the lipidated and autophagosome-associated form. The protein p62 is a particular autophagic protein and serves as a mark for autophagic flux. Calcitriol significantly reduced the LC3II/LC3I ratio in the cortex region and lowered p62 protein levels at 1–7 days compared to the TBI group (*p* < 0.05). When pretreated with chloroquine, a lysosomal inhibitor, the LC3II/LC3I ratio increased in the calcitriol group (*p* < 0.05) [39]. This study demonstrated that VDR activation lowered cell death rates caused by TBI, suggesting that VDR can act as a self-defensive protein and limit acute pathological stress in TBI. While the previous study by Tang et al. also observed that VDR expression reduced the inflammatory response caused by TBI [24], this study presented a novel finding suggesting that calcitriol can help prevent autophagy dysfunction.

Similarly, a study using vitamin D and PROG combination therapy suggested a promising avenue for combatting TBI. One study that aimed to evaluate the effectiveness of PROG and high-/low-dose vitamin D therapy on rats that had undergone TBI demonstrated this effect. In the experiment, rats were divided into sham, lesion, lesion, PROG, PROG and 1 μg/kg VDH, PROG and 2.5 μg/kg VDH, PROG and 5 μg/kg VDH groups. The rats accrued two bilateral contusions on their medial frontal cortex to mimic TBI before therapy. Twenty-one days after sustaining the injury, the rats were evaluated. All rats but those in the PROG and 5 μg/kg VDH group improved their ability to complete tasks (*p* < 0.05) compared to rats in the lesion group. Furthermore, PROG combined with 1 μg/kg of VDH provided rats with the best outcome in preserving spatial memory processing [40]. This finding presents a novel hypothesis on the benefits and drawbacks associated with varied doses of VDH, which previous preclinical studies did not mention. This suggests that vitamin D therapy is not a clear-cut solution and that improper vitamin D supplementation can be detrimental to health.

A second group studied PROG and vitamin D combination therapy on rats that underwent the same injury, bilateral contusion on the medial frontal cortex and also demonstrated the promise of combination therapy. In the study, rats were divided into the following groups: sham injury and vehicle treatment, TBI injury and vehicle treatment, TBI and PROG treatment, TBI and VDH treatment, and TBI with VDH and PROG treatment. Treatment was given intraperitoneally, both 1 and 6 h after surgery. It was discovered that combination treatment resulted in a decrease in expression of the Toll-like receptor-mediated pathway (TLR4), which plays a role in triggering the cascade of secondary damage after the initial insult (*p* < 0.01). In addition, the combination therapy also inhibited phosphorylation of NF-кB and IкBα (*p* < 0.05), which are proteins that trigger inflammatory genes [24]. Although it was shown that PROG treatment could have effects on the TLR4 and NF-кB pathway, it occurs at a later time point. When combined with VDH treatment, its effects may increase and advance to an earlier time point, halting the inflammatory cascade.

Vitamin D and omega-3 fatty acid combination therapy have also been studied in relation to TBI in mice. In the experiment, 120 rats were split into a TBI or the non-TBI group and were either given control diets or diets rich in omega-3 fatty acids and vitamin D_3_. At 48 h, 14 days, and 30 days after the initial insult, the levels of the following injury biomarkers were measured: plasma total tau (T-tau), glial fibrillary acidic protein (GFAP), ubiquitin c-terminal hydrolase L1 (UCH-L1), and neurofilament light chain (NF-L). It was found that the diet containing the omega 3-fatty acids and vitamin D increased docosahexaenoic acid levels in plasma and brain tissue (*p* < 0.05) and that the first three biomarkers mentioned (T-tau, GFAP, and UCH-L1) were shown to decrease significantly 30 days after injury [42,43,44,45,46,47,48,49]. The results from this study indicate that vitamin D combination therapy with omega-3 fatty acids can reduce inflammation and limit the onset of secondary damage.

### 3.3 Clinical investigations

Similar to the preclinical trials, clinical trials also suggest positive effects of vitamin D supplementation for those who have suffered TBI. A prospective study examined the frequency of VDH deficiency in 124 patients with TBI and found a significant correlation between VDH deficiency and the severity of the head injury and the patients’ quality of life. The study reported that VDH was significantly lower in patients with severe TBI than in patients with mild TBI. This study required the patients to fill out the Quality of Life (QoL) after brain injury questionnaire, a tool designed explicitly for patients with TBI. Self-reported scores range from 0 to 100, where 0 is the lowest QoL and 100 is the best QoL. Patients with optimal vitamin D (serum 25(OH)D concentration of 75 nmol/L or higher) scored 10.60 points higher on the questionnaire than the deficient group (serum 25(OH)D concentration of 25 nmol/L or lower) [2]. The observation did not include premorbid vitamin D status, body mass index, and other conditions in which vitamin D levels may be affected.

Jamall et al. conducted a retrospective case study and analyzed 353 patients with varying TBI severity between July 2009 and March 2015 in London. Serum 25(OH)D_3_ and parathyroid hormone concentrations were measured, and patients were divided into three groups based on their 25(OH)D_3_ concentration: deficient (<40 nmol/L), insufficient (40–70 nmol/L), and replete (>70 nmol/L). Researchers discovered that 46.5% of patients had VDD, 33.7% were insufficient, and 19.8% were replete. To measure cognitive function, Addenbrooke’s Cognitive Examination-Revised was used. Patients with VDD had lower Addenbrooke’s Cognitive Examination-Revised scores than those with VDI (*p* = 0.003) and those who were replete (*p* = 0.034). Patients with VDD had higher Beck Depression Inventory-II scores than those who had VDI (*p* = 0.003) [47]. These findings suggest that post-TBI patients have low vitamin D levels, impaired cognitive function, and exhibit more depressive symptoms. Unlike the prior study using the Endocrine Society Clinical Practice Guideline from 2011, this present study used Imperial College Healthcare NHS Trust as the guideline to categorize patients into groups based on their vitamin D levels. This demonstrates a need to standardize what values of vitamin D levels should be classified as VDD, VDI, and vitamin D-sufficient.

To observe the prevalence of VDD among patients with mild to severe TBI in acute inpatient rehabilitation, a retrospective study was performed with 369 individuals admitted between November 1, 2010, to June 30, 2015. When patients were admitted, their serum 25(OH)D levels were checked, and they were then categorized into three groups based on Clinical Practice Guidelines: deficient (<20 ng/mL), insufficient (20–29.9 ng/mL), and sufficient (≥30 ng/mL). Approximately 26% of patients were deficient, 36% were insufficient, and 39% were sufficient [48]. While previous studies suggested that VDD and VDI were associated with lower Functional Independence Measure efficiency scores, this present study found no statistical significance between the vitamin D level groups and Functional Independence Measure efficiency scores [48]. Originally, researchers had screened 798 charts but only 369 TBI patients had their vitamin D levels checked upon admission to the rehabilitation center. This discrepancy in screening might be due to the lack of standardization to check patients with TBI for VDD. Unlike the other clinical studies presented in this article, this study provides observations for vitamin D levels in acute inpatient rehabilitation, which suggests that it is important to continue monitoring vitamin D levels in post-TBI patients.

In a prospective study in India, 35 patients with moderate to severe TBI participated in a 1-year trial and were randomly placed into a treatment or placebo group. The treatment group was given a single oral dose of 120,000 IU of vitamin D (2 tablets of 60,000 IU). The placebo group was assigned 8 mg of saccharide. The patients underwent observation for the length of ICU stay. In addition, the time on mechanical ventilation, the Glasgow Coma Scale (GCS) score, and the cytokine levels of IL-6, TNFα, IFNγ, and IL-2 were monitored. The length of ICU stay and mechanical ventilation was lower in the vitamin D group than in the placebo group, at 6.19 days versus 9.07 days. The average GCS score was 3.86 units in the vitamin D group compared to the 0.19 unit decrease in the placebo group. The levels of cytokines IL-6 (*p* = 0.08), TNFα (*p* = 0.02), and IL-2 (*p* = 0.36) were lower in the vitamin D group than in the control group. The IFNγ levels improved in the vitamin D group (*p* = 0.65) compared to the placebo group [13]. Although both groups showed higher cytokine levels, the VDD group had an approximately 2- or 3-fold increase compared to the vitamin D group. IL-6 had a 5-fold increase 72 h after the initial insult, meaning that IL-6 is postulated as the main cytokine responsible for the adverse effects of the lack of vitamin D after TBI. Other studies have indicated that vitamin D can inhibit TNFα gene expression [13].

Another prospective study in India observed the prevalence of VDD in 280 patients with severe TBI admitted to the ICU from June 2017 to June 2020. Patients with a GCS score from 4 to 8 were included in the study, and their serum vitamin D level was measured upon admission. The researchers used the Endocrine Society Clinical Practice Guideline from 2011 to categorize patients with severe TBI admitted each year into three groups based on their vitamin D levels: deficient (20 ng/mL), insufficient (21–29 ng/mL), and sufficient (>30 ng/mL) [44]. Over the period of 3 years, the vitamin D-sufficient group had significantly decreased in size (*p* = 0.0001). By the third year (2019–2020), VDD in patients with severe TBI was 92% [45]. While the values of VDD increased and the rate of vitamin D sufficient patients decreased in severe TBI patients over the period of 3 years, it is important to note that much of India’s population is deficient in vitamin D [46]. This means that these findings cannot be generalized to the global population. This study emphasizes the importance of immediate vitamin D supplementation following initial insult to observe the correlations that exist between vitamin D and functional outcomes.

A prospective study observed 25(OH)D levels in 497 patients in the neurocritical care unit. VDD patients had significantly lower 3-month GOS scores, at 1 to 3, than vitamin D-sufficient patients (*p* = 0.023). In addition, patients with a low GOS score were significantly less likely to have a high phosphate level (*p* < 0.001) and more likely to be VDD (*p* = 0.023) than those who had a higher 3-month GOS score [42]. This study collected data from patients with various neurological and neurosurgical conditions and tried to control for the differences. However, this still introduced confounding variables. In future studies, it is critical to design a randomized controlled trial to determine the effects of vitamin D supplementation in the neurocritical care unit community.

Lee et al. performed a study involving 345 patients with TBI to examine the acute and long-term effects of vitamin D supplementation on patient recovery. In this study, the patients who exhibited VDD were injected intramuscularly with 100,000 IU of intramuscular cholecalciferol. As a result, the cognitive outcomes (Mini-Mental Status Examination/Clinical Dementia Rating, *p* = 0.042/*p* = 0.044) and Extended Glasgow Outcome Scale (GOS-E) (total TBI, *p* = 0.003; mild-to-moderate TBI, *p* = 0.002) significantly improved from the first week to 3 months after TBI in the patients with vitamin D supplementation [1]. The findings suggest that the provision of vitamin D in mild-to-moderate TBI patients with VDD during the acute phase of insult has the potential to improve cognitive outcomes and long-term performance. While the results of this study are promising, vitamin D supplementation was not effective in improving outcomes for patients with severe TBI. Therefore, further investigation must be done analyzing the effects of high- and low-dose vitamin D supplementation in patients with varying stages of TBI.

A double-blind, randomized, prospective study observed 74 Iranian patients with TBI. For 5 days, these patients were divided into two groups. One received a high dose of vitamin D supplementation, 100,000 IU, whereas the other received a low dose of 1,000 IU. Researchers focused on vitamin D’s effect on inflammatory markers and the mortality rate of patients with TBI. The researchers had not completed the trial at the time of publication, but they hypothesized that treating VDD in patients with TBI might help lower mortality rates and lessen inflammation. Other studies have shown that patients with TBI and VDD have increased neuroinflammation and a greater mortality rate than patients with TBI and vitamin D sufficiency [10]. Although results for this trial have not been released, this study and its methodology may facilitate future clinical trials.

In a prospective study, 3 groups consisting of 20 patients each received different treatments. In the first group, individuals were given 1 mg/kg of PROG. Those in the second group were given 1 mg/kg of PROG and 5 μg/kg of vitamin D. The third group was provided with a placebo. Before and after the treatments, the GCS means were assessed. Results showed a significant difference in the GCS mean for all three groups (*p* = 0.001). Also, the recovery rate of the group treated with PROG and vitamin D was the greatest, and the group’s mortality rate was lower than the other groups (*p* = 0.03). However, PROG (20 μmol/L) and a low dose of vitamin D (0.1 μmol/L) showed no protective effect. Conversely, PROG (20 μmol/L) and a higher dose of vitamin D (20 μmol/L) were shown to lower cell death and were more efficient than any of the drugs alone [43]. This study highlights the importance of researching combination therapy and determining the most advantageous dosage of vitamin D for patients with TBI.

## 4 Discussion

Of the potential therapeutic approaches to treat TBI, vitamin D has shown promising effects. Although the roles of vitamin D in serum calcium balance, calcium absorption, and bone metabolism are widely recognized, its essential role in the brain and CNS has only recently been appreciated. In addition, studies have found that vitamin D supplementation can improve immune performance and has significant neuroprotective and antiapoptotic functions.

VDD and VDI affect over 1 billion people around the world. Anyone with a high degree of skin melanin pigmentation, obese children and adults, pregnant women, and children and adults who do not have frequent direct sun exposure are at especially high risk [42]. Populations at risk can benefit from VDH supplementation. Unfortunately, vitamin D supplementation is not yet a widely accepted practice. This can be attributed to different cultural values, unfounded governmental and health professional apprehension, the inadequacy of information regarding natural methods of obtaining higher vitamin D levels, and the limited number of randomized controlled trials [29].

This article summarizes the current information as we progress toward understanding vitamin D’s role in modulating the secondary damage of TBI. Many of these reports have been consistent in finding that higher vitamin D levels are correlated with better outcomes. Additionally, many studies indicate that vitamin D supplementation has neuroprotective effects to combat TBI. Studies also show that vitamin D supplementation leads to better functional outcomes, such as improved GOS-E scores. Furthermore, it has been shown that determining cytokine levels after TBI is crucial to understanding how to prevent secondary injury.

Although the current research has been valuable, limitations are present. First, dosing, time to treatment, and the duration of treatment should be standardized. Second, testing for potential gender differences in response to VDH therapy is important. Some studies primarily focused on males, and it is well-established that hormonal fluctuations could have significant consequences for treatment outcomes. Third, the cognitive function of individuals diagnosed with severe TBI is difficult to assess, so data collection relies primarily on those admitted with mild to moderate TBI. Finally, in several studies, some patients did not have their vitamin D levels checked within 24 h of admission. Thus, the lack of data makes it difficult to draw reliable conclusions.

This article adds to the current knowledge of vitamin D and TBI by summarizing the benefits of vitamin D supplementation on TBI, the possibility of combination therapy as a method of treating TBI, and the risk factors associated with both VDD and TBI, as well as the various mechanisms of vitamin D and TBI.

## 5 Conclusions

Research on the role of vitamin D in TBI remains a promising avenue. The studies described here suggest that vitamin D could be a modifiable risk factor to improve recovery after TBI. Although several studies have been conducted, more research is needed to observe the longitudinal effects of vitamin D status and supplementation on neurological function and levels of inflammatory biomarkers after TBI. In addition, it is important to focus on standardizing TBI trials to ensure that more meaningful data are gathered. It is also crucial to discover more mechanisms associated with vitamin D and TBI to treat and potentially prevent secondary insult. Further research can better assess whether the treatment of VDD and/or supplementation should become part of clinical guidelines in the management and care of TBI. Additionally, combinational therapies might help provide a better outcome for patients with TBI and, thus, should continue to be researched.

## 6 Literature search

The information used for this literature review was accumulated by using search engine tools such as Google Scholar, the University of Florida library PRIMO database, Embase, and PubMed. Various keywords of “Vitamin D” AND “TBI” were used to ensure all results were covered. Terms for vitamin D included “Vit D”, “ergocalciferol”, “calciferol”, “25 hydroxyvitamin D”, and “calcifediol”. Terms for TBI included “traumatic brain injury”, “trauma”, “concussion”, and “brain injury”. Articles not written or translated in English were excluded.
